# Point-of-Care Ultrasound Optimizes the Preoperative Use of Prostaglandin E1 in Infants With Transposition of the Great Arteries

**DOI:** 10.31083/RCM39250

**Published:** 2025-08-27

**Authors:** Wei Zhang, Yu-Yu Tan, You-Qun Zou, Shu-Sheng Wen, Min Yang, Yu-Mei Liu

**Affiliations:** ^1^Department of Neonatology, Guangdong Provincial People’s Hospital (Guangdong Academy of Medical Sciences), Southern Medical University, 510080 Guangzhou, Guangdong, China; ^2^Department of Cardiovascular Surgery, Guangdong Cardiovascular Institute, Guangdong Provincial People’s Hospital, Guangdong Academy of Medical Sciences, 510080 Guangzhou, Guangdong, China; ^3^Department of Pediatrics, Guangdong Provincial People’s Hospital (Guangdong Academy of Medical Sciences), Southern Medical University, 510080 Guangzhou, Guangdong, China

**Keywords:** transposition of the great arteries (TGA), patent ductus arteriosus (PDA), prostaglandin E1 (PGE1), point-of-care ultrasound (POCUS), pulse oximetry saturation (SpO_2_)

## Abstract

**Background:**

This study aimed to determine the optimal dosages of prostaglandin E1 required to maintain a patent ductus arteriosus (PDA) in infants with transposition of the great arteries (TGA) based on point-of-care ultrasound (POCUS) findings.

**Methods:**

Infants with TGA were recruited from two groups (the historical control group and the POCUS group that received POCUS in combination with pulse oximetry saturation (SpO_2_) to titrate the dose of prostaglandin E1 (PGE1)).

**Results:**

A total of 150 patients were included in this study. The mean gestational ages were 38.6 weeks and 38.9 weeks, respectively, and the mean birth weights were 3.09 kg and 3.23 kg, respectively, in the control and POCUS groups. The rate of PGE1 prescriptions in the control group (93.3%) was higher than in the POCUS group (71.1%; *p* < 0.001). The time at which PGE1 was initiated (prenatally diagnosed) was earlier than in the control group (0.05 ± 0.01 vs. 1.66 ± 3.72 d; *p* < 0.001). The proportion of patients using a low dose (less than 5 ng/kg⋅min) of PGE1 was higher in the POCUS group (40.6% vs. 8.9%; *p* < 0.001). The multivariate logistic regression analysis indicated that implementing POCUS significantly reduces the dosage of PGE1.

**Conclusion:**

POCUS can optimize the use of PGE1, reduce unnecessary usage, postpone the initiation of PGE1, minimize the maintenance dose, and reduce the impact dose. POCUS guidance enhances the safety and effectiveness of PGE1 in infants with TGA.

## 1. Introduction

Dextro-transposition of the great arteries (TGA) is one of the most common cyanotic congenital heart defects, with an incidence of approximately 300 per million live births [1]. The arterial switch operation (ASO), first described by Adib Jatene in 1976 [2], currently represents the procedure of choice [3, 4]. The crucial factor that influences the natural history of TGA is the presence of mixing lesions, such as an atrial septal defect (ASD), a ventricular septal defect (VSD), or a patent ductus arteriosus (PDA). In TGA with an intact ventricular septum (TGA/IVS), atrial-level mixing can be supplemented through maintaining an adequate PDA, with the use of prostaglandin. In cases of TGA with VSD, intracardiac mixing is often sufficient, and therefore, maintaining ductal patency may not be necessary. Meanwhile, it is advisable to initiate prostaglandin E1 (PGE1) for patients with cyanosis. To maintain ductal patency, the standard doses range from 10 to 50 ng/kg⋅min, and patients can be tapered off starting 2–4 hours after initiation, provided that pulse oximetry saturation (SpO_2_) and tissue perfusion remain acceptable [4, 5, 6, 7, 8]. Nonetheless, the use of PGE1 might not be suitable, as ductal shunting is frequently insufficient in the presence of a restrictive interatrial communication, or, because the PGE1 dose is too large, opening the PDA may also result in pulmonary overcirculation. Currently, no standard clinical guide exists for determining the appropriate dose for an individual patient.

The risks of apnea, hypoventilation, fever, neurological side effects, necrotizing enterocolitis, and cortical hyperostosis are noted as the side effects of PGE1 therapy [9, 10, 11]. There is evidence suggesting that the respiratory depression induced by PGE1 is dose-dependent [12]. Furthermore, persistent left-to-right shunting across the ductus arteriosus might cause pulmonary edema, which could affect patient stability and require escalation of therapy and airway support. Occasionally, infants with complex TGA might have other cardiac malformations or might be complicated by persistent pulmonary hypertension of the newborn (PPHN) [13]. In such cases, even when high doses of PGE1 are administered, these infants might develop refractory cyanosis that requires urgent surgical correction. The side effects and improper use of PGE1 may exacerbate the conditions of the patients and the requirement for mechanical ventilation and neonatal intensive care. Therefore, utilizing lower doses of PGE1 has been explored to minimize the adverse effects of PGE1 while maintaining its efficacy [14, 15, 16].

Point-of-care ultrasound (POCUS) has become an essential tool for clinicians, similar to a stethoscope [17]. POCUS assessment of PDAis a convenient and reliable method. Indeed, the implementation of POCUS can monitor the ductal size, shape, and shunt daily. In addition, POCUS can be used to assess cardiac function.

We hypothesized that POCUS combined with SpO_2_ can optimize the preoperative use of PGE1 in infants with TGA/IVS. Therefore, this study primarily aimed to investigate the optimal use of PGE1, including the rate of administration, dose, and duration of treatment. The secondary objective was to assess whether reducing the use of PGE1 resulted in reduced treatment efficacy, increased risk of perioperative death, and any differences in adverse effects.

## 2. Materials and Methods

This was a single-center, non-randomized, historical, controlled study conducted at a third-level NICU. Ethical approval was obtained from the Institutional Review Board of our hospital (IRB number: KY2023-737-01).

Infants from two periods were included in our study. Infants in Epoch 1 (from January 1, 2010, to December 31, 2013) were assigned to the historical control group, without POCUS-guided PGE1 dose titration before ASO. Infants in Epoch 2 (from January 1, 2017, to December 31, 2022) were assigned to the observation group (POCUS group), with POCUS combined with SpO_2_ to guide PGE1 titration before ASO. When setting up the POCUS program in our NICU, we gradually incorporated POCUS into our approach, using it in combination with SpO_2_ to guide PGE1 titration. By 2016, this program had become a routine practice to titrate preoperative PGE1 in infants with TGA.

### 2.1 Inclusion and Exclusion Criteria

Inclusion criteria: Infants with TGA/IVS younger than 3 months of age who underwent ASO in our hospital.

Exclusion criteria: We excluded patients who had been treated with PGE1 for more than 48 hours at other hospitals before admission, since the doses administered before transport were often poorly recorded and used empirically.

### 2.2 PGE1 Protocol

#### 2.2.1 Epoch 1 (the Control Group)

Infants born in our hospital with a prenatal diagnosis of TGA were administered PGE1 immediately after admission to the NICU. The starting dose of PGE1 was determined based on the SpO_2_ level. PGE1 was titrated according to the target SpO_2_ range of 75% to 85% [18]. The initial PGE1 dose was 1–5 ng/kg⋅min when the SpO_2_ was over 85%, 5–10 ng/kg⋅min when the SpO_2_ ranged from 75% to 85%, and 10–50 ng/kg⋅min when the SpO_2_ was below 75%. An echocardiogram was performed in the early hours after birth, and the PGE1 dose is adjusted according to the SpO_2_ level to maintain a SpO_2_ range of 75–85%. If SpO_2_ was persistently above 85%, the PGE1 dose was gradually reduced or withdrawn. If SpO_2_ was below 65% or accompanied by elevated lactate, a short-term impact dose (20–100 ng/kg⋅min) would be considered. The usage strategy at that time was based on relevant guidelines and literature, combined with the clinical condition of the patient, as established by the department [5, 19].

For patients transferred to our hospital with a postnatal diagnosis, PGE1 was initiated upon confirmation of the diagnosis, either upon or after admission, depending on the availability of PGE1. After initiation, PGE1 would be titrated in accordance with the protocol described above for the infants with the prenatal diagnosis.

#### 2.2.2 Epoch 2 (the POCUS Group)

No universally fixed size currently exists for a PDA [18]. In the clinical practice of our department, it was observed that many neonates with restrictive interatrial communication required a thicker PDA to maintain oxygenation, and a diameter of less than 2.5 mm consistently failed to provide sufficient mixing. Notably, larger PDAs (>4 mm) risk pulmonary overcirculation and systemic steal. For infants who require PGE1 to maintain a PDA, we set a target PDA size of 2.5–3.5 mm to sustain a SpO_2_ of 75–85%. POCUS was conducted to monitor the size of the PDA, along with SpO_2_ monitoring, to achieve the target PDA size and SpO_2_ levels. When SpO_2_ was above 85%, routine monitoring of the PDA was not required, and the use of PGE1 was suspended. When SpO_2_ was within the range of 75–85%, the size of the PDA was monitored daily using POCUS. When signs of constriction or a diameter of the PDA were less than 2.5–3.5 mm, PGE1 was initiated at a rate of 1–5 ng/kg⋅min. PGE1 was reduced and withdrawn when the diameter of the PDA exceeded 4–4.5 mm or the oxygen saturation was consistently over 85%. When the SpO_2_ level was within the range of 70–75%, we monitored the size of the PDA using POCUS and administered PGE1 at a rate of 5–10 ng/kg⋅min, while also evaluating the need for increased respiratory support, including oxygen supplementation or non-invasive positive pressure ventilation. After administering PGE1, when the SpO_2_ was within the range of 75–85% with a PDA diameter of 2.5–3.5 mm, PGE1 was titrated to maintain the SpO_2_ above 75% at the minimum dose. If the SpO_2_ was consistently below 75% with a PDA diameter less than 2.5 mm, PGE1 would be titrated at 10 to 50 ng/kg⋅min. During this period, it was crucial to assess the need for enhanced respiratory and circulatory support, the presence of PPHN, and the potential requirement for urgent surgical intervention. Although the PGE1 dose adjustment involved multi-parametric homeostasis, this strategy aimed to individualize responses to the dynamics of the neonatal circulation. We minimized subjectivity by using standardized protocols and team-based decision-making.

### 2.3 Bedside POCUS

Bedside POCUS was performed by neonatologists with considerable experience in using a pulsed-wave Doppler *(Mindray, Model 9T)* equipped with a 3–7 MHz shallow-focus transducer. The PDA was imaged from the left parasternal position using a direct inferior or slightly superior position, and the minimum diameter was measured through frame-by-frame analysis. If the entire PDA could not be visualized through a standard parasternal approach, an alternative position was adopted, with the probe placed just below the right or left clavicle. The probe was rotated to align with the long axis of the aortic arch. Subsequently, the probe was turned downwards to visualize the PDA, the main pulmonary artery, and the aortic arch in the same section [20, 21]. The size of the PDA was assessed by measuring the minimum and maximum intraluminal diameters using two-dimensional echocardiography. The smallest measurement obtained at the pulmonary end of the ductus arteriosus, as determined by color-Doppler mapping, was defined as the ductal diameter [22]. An inner ductal diameter of less than 2 mm, as described above, was regarded as the first sign of ductal constriction [9, 23]. The PDA diameter was measured using echocardiography by two independent neonatologists trained in bedside ultrasound, with the average of three consecutive cardiac cycles recorded.

### 2.4 Clinical Parameters

Baseline clinical characteristics, such as gender, gestational age, birth weight, and prenatal diagnosis, were documented. Medication records were reviewed to determine whether the patient had been transported on PGE1. We documented the total duration of PGE1 administration and any discontinuation or change in dose. PGE1 treatment success was defined as achieving a SpO_2_ level within 70–85% before surgery. Due to the complexity and variability of the data, the patients were divided into two groups for analysis based on the dose of PGE1: Low-dose group (the PGE1 dose was less than 5 ng/kg⋅min) and high-dose group (the PGE1 dose was higher than 5 ng/kg⋅min or requiring the use of an impact dose of 20–100 ng/kg⋅min). More detailed groupings were conducted based on the dosage used, from smallest to largest as follows: Group A, the PGE1 dose was rapidly reduced and stopped within 24 hours; Group B, the PGE1 dose was maintained at 1–5 ng/kg⋅min without discontinuation; Group C, the PGE1 dose was 5–10 ng/kg⋅min; Group D, with a dose exceeding 10 ng/kg⋅min or requiring the use of an impact dose of 20–100 ng/kg⋅min.

Seizures, respiratory depression, fever, and necrotizing enterocolitis (NEC) were recorded, and these conditions were carefully analyzed for the potential for adverse effects of PGE1. The need for enhanced respiratory support and/or caffeine for treating apnea was noted. When the PGE1 dose exceeded 10 ng/kg⋅min, respiratory depression was particularly common. Caffeine was prescribed to decrease these risks [9, 11, 24, 25].

### 2.5 Statistical Analysis

Descriptive statistics were employed to analyze the demographic and morbidity data. The normal distribution and equal variance were verified for continuous variables. The Shapiro–Wilk test was utilized to determine whether the numeric variables were normally distributed. Normally distributed data are presented as the mean ± standard deviation. Non-normally distributed data are presented as the median and the interquartile range. Differences among categorical variables were examined via Pearson’s chi-squared test or Fisher’s exact test. Differences in means between normally distributed continuous variables were assessed using the Student’s *t*-test or analysis of variance. We used logistic regression to estimate the association of POCUS with the dosage of PGE1. Variables with a value of *p* < 0.1, as well as factors potentially associated with PGE1 administration, were included in the multivariate logistic regression analysis. The results are presented as odds ratios (OR) along with their corresponding 95% confidence intervals (CIs). All analyses were conducted using the IBM SPSS® Version 23.0 software (SPSS Inc., Chicago, IL, USA). Statistical significance was defined as *p* < 0.05 for all analyses.

## 3. Results

A total of 60 patients were included in the control group (Epoch 1) and 90 patients in the POCUS group (Epoch 2). The clinical characteristics are summarized in Table 1. Demographic information and clinical characteristics at baseline were almost similar. However, the rate of prenatal diagnosis and patient referrals differed significantly between the two groups (both *p* < 0.001).

**Table 1.** **Clinical and baseline characteristics**.

Study cohort	Epoch 2 (n = 90)	Epoch 1 (n = 60)	*p*-value
Male gender, n (%)	77 (85.6)	50 (83.3)	0.711
Birth weight (mean ± SD), kg	3.09 ± 0.42	3.23 ± 0.43	0.055
Gestational age (mean ± SD), week	38.6 ± 1.6	38.9 ± 1.1	0.121
Apgar score <7 at 1 min, n (%)	9 (10.0)	2 (3.3)	0.224
Caesarean section, n (%)	39 (43.3)	23 (38.3)	0.542
Referral patient, n (%)	42 (46.7)	55 (91.7)	<0.0001
Prenatal diagnosis, n (%)	57 (63.3)	5 (8.3)	<0.0001
Prescribe PGE1, n (%)	64 (71.1)	56 (93.3)	0.001
Emergency surgery, n (%)	33 (36.7)	33 (55.0)	0.030
Age at operation (mean ± SD), d	8.8 ± 7.5	12.9 ± 11.7	0.018
Death after operation, n (%)	4 (4.4)	7 (11.7)	0.179

Epoch 1: The control group, admitted from January 1, 2010, to December 31, 2013. Epoch 2: The POCUS group, admitted from January 1, 2017, to December 31, 2022. Referral patient: Those transferred from other medical facilities, including hospitals of all levels. Prescribe PGE1: PGE1 was used, regardless of dose and duration. Emergency surgery: required within 1 day of birth or for acute, life-threatening conditions, including a significant increase in lactate, persistent hypoxemia. PGE1, prostaglandin E1.

### 3.1 The Utilization of Data and Efficiency of PGE1

The rate of PGE1 use in the control group (93.3%) was significantly higher than in the POCUS group (71.1%; *p* < 0.001). The initial initiation time of PGE1 use in the control group (prenatally diagnosed) was earlier than in the POCUS group (0.05 ± 0.01 d vs. 1.66 ± 3.72 d; *p* < 0.001). The proportion of patients using low-dose PGE1 in the POCUS group was 40.6%, which was significantly higher than the 8.9% noted in the control group. The dose grouping ratios between the POCUS group and the control group were as follows: Group A (10.9% vs. 1.8%), Group B (29.7% vs. 7.1%), Group C (40.6% vs. 60.7%), and Group D (18.8% vs. 30.4%). All of the above were statistically significant differences (*p* < 0.001; Table 2). There was no difference in the improvement of SpO_2_ after using PGE1 between the two groups (*p* = 0.404; Table 2). The requirement for emergency surgery decreased in the POCUS group (*p* = 0.030; Table 1).

**Table 2.** **The usage data and efficiency of PGE1**.

Study cohort		POCUS (n = 64)	The control group (n = 56)	*p*-value
Time for initiating PGE1 (mean ± SD), d (prenatal diagnosis) *		1.66 ± 3.72	0.05 ± 0.01	<0.0001
SpO_2_ before using PGE1 (mean ± SD, %)		59.05 ± 18.08	63.38 ± 16.88	0.180
SpO_2_ after using PGE1 (mean ± SD, %)		72.78 ± 13.78	74.82 ± 14.52	0.432
Change in saturation (mean ± SD, %)		13.73 ± 16.46	11.47 ± 12.97	0.404
Low dose (less than 5 ng/kg·min), n (%)		26 (40.6)	5 (8.9)	<0.0001
PGE1 dosage group, n (%)				<0.0001
	A. Reduced and stopped within 24 hours	7 (10.9)	1 (1.8)	
	B. 1–5 ng/kg·min without discontinuation	19 (29.7)	4 (7.1)	
	C. 5–10 ng/kg·min	26 (40.6)	34 (60.7)	
	D. Exceeding 10 ng/kg·min	12 (18.8)	17 (30.4)	

*: Neonates with a prenatal diagnosis of TGA, the time of initiation of PGE1 in both groups. Change in saturation: changes in saturation after using PGE1. PGE1 dosage group: The study was divided into four groups based on the fluctuation range of the PGE1 dose: Group A, Group B, Group C, and Group D. TGA, transposition of the great arteries; POCUS, point-of-care ultrasound.

### 3.2 Respiratory Depression and Fever During the Use of PGE1

In infants receiving PGE1, there was no significant difference in the incidence of fever (*p* = 0.923) or respiratory depression (*p* = 0.697; Table 3) between the two groups. In Epoch 1, two cases in Group D and three cases in Group C developed respiratory depression, while five cases in Group D, three cases in Group C, one case in Group B, and one case in Group A developed a fever. In Epoch 2, one case in Group D, three cases in Group C, and two cases in Group B developed respiratory depression, while five cases in Group D, four cases in Group C, and two cases in Group B developed a fever (Table 3).

**Table 3.** **Respiratory depression and fever observations under the use of PGE1**.

Study cohort		POCUS (n = 64)	The control group (n = 56)	*p*-value
Respiratory depression, n				0.697
	No	26	27	
	Unknown^a^	32	24	
	Yes	6	5	
Fever, n				0.923
	No	53	46	
	Yes	11	10	

^a^ A ventilator was required to support treatment due to illness before or during the use of PGE1.

### 3.3 Logistic Regression Analysis of PGE1 Dosage

Consistent with contemporary methodological guidance (STROBE Statement 2007 [26]; Lee S 2017 [27]), our variable selection process explicitly balanced statistical criteria with domain knowledge derived from neonatal cardiac surgery studies (Kumar 2021 [28]). Variables with a value of *p* < 0.1 from the univariate analysis, as well as those potentially associated with PGE1 administration, were included as independent variables in the multivariate logistic regression analysis using the Wald test. The results indicated that the use of POCUS significantly reduces the dosage of PGE1. Detailed findings are presented in Table 4.

**Table 4.** **Logistic regression analysis of PGE1 dosage**.

Variable	β	Wald	*p*-value	OR (95% CI)
POCUS	1.394	5.013	0.025	4.03 (1.19, 13.65)
Birth weight	–0.169	0.071	0.790	0.845 (0.244, 2.93)
Gestational age	0.108	0.328	0.567	1.114 (0.77, 1.62)
Apgar score <7 at 1 min	1.262	2.730	0.098	3.533 (0.79, 15.79)
Prenatal diagnosis	–0.249	0.043	0.835	0.780 (0.075, 8.103)
Referral patient	–0.668	1.240	0.266	0.513 (0.16, 1.66)
Intercept	–4.227	0.327	0.568	0.015

The β-value of POCUS is large, with a value of *p* < 0.05, which has a positive impact on the application of PGE1.

## 4. Discussion

The use of PGE1 is sometimes indispensable and life-saving for children with cyanotic congenital heart disease. Adverse events observed in the literature are common and include apnea, pyrexia, and hypokalemia [7, 9, 29]. We have presented a report from a prominent tertiary neonatal unit in China regarding the integration of POCUS into routine clinical practice for the preoperative treatment of children with TGA. We found a significant reduction in the number of infants receiving PGE1 after POCUS. Indeed, POCUS could reduce the dosage of PGE1 and delay the age at which PGE1 is initiated. The safety and effectiveness of this method were established.

### 4.1 The Status of PGE1 Use

Preoperative use of PGE1 in infants with TGA depends on the individual clinical indication. An intravenous (IV) infusion of PGE1 is recommended immediately after birth, until postnatal echocardiograms are completed and all forms of inter-circulatory mixing have been evaluated. PGE1 has been employed in diverse dosing regimens: a higher dose of up to 100 ng/kg⋅min might be required when the ductus needs to be reopened [4, 5]. To maintain ductal patency, standard doses range from 10 to 50 ng/kg⋅min. Patients can be tapered off starting 2–4 hours after initiation, provided that SpO_2_ levels and tissue perfusion remain acceptable [4, 5, 6, 7, 8].

### 4.2 Optimization of the PGE1 Dose

#### 4.2.1 Minimize Unnecessary Use

Infants diagnosed prenatally usually initiate their infusion shortly after birth, and the dose is then adjusted according to the clinical situation and evaluation of cardiac color Doppler ultrasound. In the POCUS group, 57 out of 90 TGA/IVS infants with a prenatal diagnosis were identified, among whom only 38 cases received PGE1. The initial infusion time was 1.66 ± 3.72 days, and 26 cases were on low-dose maintenance. This study demonstrates that the use of PGE1 can be adjusted with greater confidence using POCUS guidance. For newborns with stable circulation and no severe cyanosis, the application of PGE1 can be postponed or even omitted.

#### 4.2.2 Reduction of the PGE1 Dose

Currently, some studies and retrospective chart reviews with small patient numbers have reported that doses lower than the manufacturer’s suggested dosing protocol can be used to effectively maintain a PDA [16, 30, 31]. Three studies published in the 1980s and 1990s reported that a lower initial dose of PGE1 (mean dose, 5–10 ng/kg⋅min) could be used successfully to maintain a PDA [12, 29, 31]. Yucel *et al*. [30] provided evidence that maintenance doses as low as 3–5 ng/kg⋅min are a safe and effective therapy for critical CHD in their cohort of 154 patients. Gordon *et al*. [16] showed PGE1 therapy was effective in maintaining the PDA in neonates with low-dose (less than 10 ng/kg⋅min) regimens in 75 patients. In our study, the proportion of PGE1 in the POCUS group was 71.1%, and nearly half (26/64, 40.6%) of the children received a low-dose (less than 5 ng/kg⋅min) of PGE1. Among them, seven cases (10.9%) were rapidly reduced within 24 hours, while the maintenance dose in 19 cases (29.7%) was between 1 and 5 ng/kg⋅min, which was lower than in other studies and the control group in our study.

The effectiveness of PGE1 has been based on the clinical condition of the infant, arterial blood gas analysis, improvements in oxygen saturation, acidosis, vital signs, and PDA size [4, 31]. In our study, there were no significant differences in SpO_2_ improvement and perioperative mortality after using PGE1. The overall emergency surgery rate was significantly reduced (*p* = 0.03). Our findings suggest that the PGE1 dose can be optimized with POCUS guidance, ensuring both safety and efficacy. Meanwhile, strategies for prenatal diagnosis and the integrated management of infants with CHD have been effective in recent years. The tertiary hospital equipped with specialized treatment facilities empowers newborns to receive a superior level of standardized and timely perioperative management for CHD. Further, research has demonstrated that the duration of treatment is a risk factor that correlates with dose escalation [30]. Furthermore, it was noted that pausing and resuming the PGE1 infusion did not appear to be a risk factor for increasing doses [12, 30]. This study substantiated this finding, illustrating that individuals whose PGE1 infusion was paused and resumed did not exhibit higher rates of increased doses (5/17, 29%) compared to those who received continuous infusions [32]. The time to the initial PGE1 treatment has been demonstrated to correlate with an increased dosage [12].

#### 4.2.3 The Significance of Optimizing the Utilization of PGE1

In this study, we found that PGE1 maintenance time might be associated with the optimal timing of surgery or a change in the condition of the patient as assessed by the surgeon. By using the impact dose, POCUS can guide the adjustment of PGE1, determining whether to continue increasing or decreasing the dose, considering the presence of PPHN, and immediately proceeding with emergency surgery.

We also focused on the circumstances of infants who required emergency surgery. In the POCUS group, a total of eight cases required emergency surgery due to a significant disparity in SpO_2_ levels and severe heart failure. The majority of these cases were considered to be associated with PPHN. For these children, it was inadvisable to increase the dosage of PGE1 indiscriminately and attempt to enlarge the PDA [4]. In such cases, more effective anti-heart failure treatments, such as nitric oxide therapy, and even controlling the size of the PDA, were often necessary. Moreover, immediate ASO is mandatory if the condition remains irreversible [13, 33]. In this study, we also found that in four cases involving low-dose PGE1, the PDA was too large and resulted in excessive pulmonary blood flow, and it was necessary to discontinue PGE1 promptly. Therefore, it is critical to optimize the use of PGE1 in TGA infants.

### 4.3 Adverse Effects of PGE1

Adverse drug events of PGE1 included apnea, tachypnea, bradycardia, tachycardia, hypotension, hypokalemia, hyperkalemia, hypocalcemia, fever, tremors, bleeding, edema, neurologic side effects, necrotizing enterocolitis, and cortical hyperostosis [8, 9]. Since our study was retrospective, some clinical symptoms were not fully recorded. The adverse effects of PGE1 focused mainly on respiratory depression and fever.

At an initial dose of 25 to 50 ng/kg⋅min, apnea occurred in 42%, while intubation due to respiratory depression occurred in 14% of infants as reported by Tálosi *et al*. [19]. Other studies have explored lower doses of PGE1 and reported a lower incidence of respiratory depression [30, 34]. There is evidence indicating that respiratory depression induced by PGE1 is dose-dependent [12, 35], although one study contradicts this finding [29]. However, we could not confirm that a lower dose of PGE1 could reduce the risk of respiratory depression (9.4% vs. 8.9%; *p* = 0.697). Our cohort reported an overall rate of clinically relevant respiratory depression of 9.2%, and all these patients required mechanical ventilation due to respiratory depression. The accurate assessment of the risk of respiratory depression had been hindered since nearly half of the children required non-invasive or mechanical ventilation as a result of their condition either before or after PGE1 usage. When administering PGE1 at a dosage exceeding 10 ng/kg⋅min, it was advisable to use caffeine to prevent apnea. Moreover, we found that the children in the control group of this study were more often transferred from other hospitals and were older at the time of diagnosis. The initiating use of PGE1 might indicate a stronger tolerance to PGE1. This suggests that there are many confounding factors for the dosage of PGE1, and that studies with larger sample sizes are needed. We found that the overall rate of clinically relevant fever was 17.5% in our study. There was no significant difference between the POCUS group and the control group (17.2% vs. 17.9%; *p* = 0.095).

Our data found that even low-dose (less than 5 ng/kg⋅min) PGE1 was at risk of respiratory depression, which may be affected by preterm birth, low birth weight, the early postnatal period, and cardiac insufficiency. This is consistent with other studies [16, 30]. Therefore, regardless of whether the side effects of PGE1 are dose-dependent, a lower dose of PGE1 does not appear to increase the risk of respiratory depression or fever.

### 4.4 Feasibility of Universal Use of POCUS

POCUS is a non-invasive, low-risk imaging modality that can be used to diagnose and help guide the management of critically ill children in the cardiac intensive care unit. POCUS can be performed by an intensivist at the bedside of patients with real-time interpretation, leading to rapid clinical decision-making and the potential to improve patient outcomes [36]. Recent studies support the use of POCUS for accurately assessing left ventricular systolic function, diagnosing pericardial effusion, pulmonary embolism, identifying pulmonary edema and pneumonia, as well as consensus statements on the use of cardiac and lung POCUS in clinical practice [37, 38]. The Society of Point of Care Ultrasound (SPOCUS) formed a working group in 2022 to establish a set of recommended best practices for POCUS, applicable to clinicians regardless of their training, specialty, resource setting, or scope of practice [39]. However, achieving real-time bedside monitoring of PDA dimensions to guide pharmacological interventions and clinical decision-making remains a significant challenge in pediatric patients with cyanotic congenital heart disease. The training program for senior residents at our institution spans a duration of 1 to 3 years. Will such training face significant challenges? A study described the national state of POCUS training in residency programs and evaluated the implementation of the core POCUS curriculum in Canada [40]. POCUS leaders believe their residents are proficient in the core POCUS applications by the end of training, except for advanced cardiac and thoracic ultrasound. It is believed that senior doctors from high-level centers can meet the training requirements and widely apply POCUS to provide better clinical strategies for patients.

## 5. Limitations

This study involved a retrospective chart review without randomized dosing. The observational comparative study design contained inherent statistical limitations. However, since TGA was a rare event, the sample size might have been insufficient to detect alterations in adverse events. Moreover, the dose and maintenance time of PGE1 for patients referred from other hospitals were often incomplete, and the timing of use may not have been accurate. There was no specific delineation of the dosage and changes of PGE1 for each child, and the involved infants were grouped according to dose range. The study period spanned a long duration, and certain disparities existed between the two study populations, including the rate of prenatal diagnosis and referral situations. There were variances in the indications for surgical evaluation, surgical techniques, and perioperative nursing and treatment techniques. These differences could lead to modifications in emergency surgical evaluation criteria and postoperative rehabilitation protocols.

Considering the above limitations, it is essential to ensure the completeness of clinical data and the uniformity of treatment plans in future research protocols. In terms of statistical analysis of the study, more groupings should be conducted, including birth weight, gestational age, transfer treatment, other cardiac malformations, genetic lesions, asphyxia resuscitation, and pneumonia. Addressing missing PGE1 data requires a dual approach: Employing robust statistical methods to address existing gaps and establishing proactive infrastructure for future studies. By combining imputation techniques with EHR integration, training, and real-time monitoring, researchers can ensure the collection of high-quality data for evaluating the safety and efficacy of PGE1. Future studies should prioritize interoperable systems and pragmatic designs to minimize missingness from the outset.

## 6. Conclusion

Bedside point-of-care ultrasound, in combination with SpO_2_, can optimize the utilization of PGE1 by reducing unnecessary usage, postponing the initial utilization time, minimizing the maintenance dose, and lowering the impact dose. We acknowledge that not all patients with TGA are classically ductal-dependent and may not uniformly benefit from PGE1. Nonetheless, POCUS can be easily implemented in tertiary neonatal units. POCUS guidance allows safe reduction of PGE1 dosage and delays initiation in TGA/IVS infants.

## Availability of Data and Materials

The data sets generated and analyzed during the current study are not publicly available due to privacy or ethical restrictions (containing sensitive personal health information) but are available from the corresponding author on reasonable request.
